# Occurrence and prevalence of bacteria on door handles at the University of Port Harcourt Teaching Hospital and the multidrug resistance implications

**DOI:** 10.1099/acmi.0.000615.v4

**Published:** 2023-07-12

**Authors:** Deinmo Edi, Ovinuchi Ejiohuo, Best Ordinioha

**Affiliations:** ^1^​ The Department of Preventive and Social Medicine, Faculty of Clinical Sciences, University of Port Harcourt, Port Harcourt, Rivers State, Nigeria; ^2^​ Department of Pharmacognosy and Biomaterials, Poznan University of Medical Sciences, Poznan, Poland; ^3^​ Doctoral School, Poznan University of Medical Sciences, Poznan, Poland

**Keywords:** multidrug-resistant bacteria, infectious disease, antimicrobial resistance, microbial assessment, HCAI, MDRO, public health

## Abstract

**Background.** The coronavirus disease 2019 (COVID-19) pandemic has heightened concerns regarding transmitting hospital-acquired infections through high-contact points in healthcare facilities, such as door handles, which can serve as reservoirs for pathogenic micro-organisms. With the increased infectivity of the serious acute respiratory syndrome coronavirus 2 (SARS-CoV-2) virus, there is greater awareness of the risk of multidrug-resistant bacteria on these surfaces, necessitating strict surveillance prevention protocols.

**Objective.** This study assesses the occurrence and prevalence of bacteria on toilet door handles at the University of Port Harcourt Teaching Hospital in Nigeria and the multidrug resistance implications. We examined the hospital infection level and overall sanitary conditions.

**Method.** Experimental data from our study on hospital door handles in different locations were assessed. Eighty-six samples were collected from toilet door handles at the University of Port Harcourt Teaching Hospital. Ethical approval was obtained from the hospital departments and the university research ethics committee. Samples from private offices and doors without handles were excluded. Bacteria were isolated from the samples, and antibiotic sensitivity testing was performed.

**Result.** Of the 329 bacteria isolated, 55.5 % were Gram-positive and 44.4 % Gram-negative. The most prevalent bacteria were *Staphylococcus epidermidis *, followed by *Bacillus* sp. The prevalence of multidrug-resistant bacteria was also low, with *Enterococcus* sp. having the highest prevalence of 28.57%, followed by *Micrococcus* sp. at 3.45 % and *S. epidermidis* at 1.96 %.

**Conclusion.:**

The results show that, despite the occurrence of pathogenic micro-organisms, there has been a reduction in the prevalence of multidrug-resistant bacteria strains. This study can be used as an example for environmental microbiological surveillance in suspected outbreak investigations and assessment of sanitary conditions and the prevalence of multidrug-resistant bacteria in healthcare facilities.

## Data Summary

The authors confirm that all supporting data and protocols have been provided within the article.

**Impact Statement**

This study highlights the importance of maintaining and encouraging certain key aspects of hospital coronavirus disease 2019 (COVID-19) protocols, such as constant disinfection of surfaces, hand hygiene and contactless doors, to limit the spread of hospital-acquired diseases. These practices should be standard in healthcare facilities to prevent the transmission of nosocomial diseases, particularly those caused by multidrug-resistant bacteria. Overall, this study underscores the significance of environmental microbiological surveillance in healthcare facilities during suspected outbreaks to ensure sanitary conditions and checkmate the prevalence of multidrug-resistant bacteria. The findings can guide policymakers and healthcare professionals in implementing effective infection prevention and control measures to promote public health and safety.

## Introduction

The coronavirus disease 2019 (COVID-19) pandemic has significantly influenced how hospital-acquired infections are spread because of the large number of hospitalizations, resulting in multiple people touching different surfaces in healthcare facilities. High-contact points in healthcare facilities refer to areas or surfaces frequently touched by multiple people, such as door knobs, handles and railings. These surfaces can act as reservoirs for pathogenic micro-organisms that can cause infections and diseases [1]. The presence of these micro-organisms on high-contact points can pose a risk to public health because they can be transmitted from one person to another through direct contact or by touching contaminated surfaces and then touching the eyes, nose, or mouth.

Since serious acute respiratory syndrome coronavirus 2 (SARS-CoV-2 virus) is mostly transmitted by respiratory droplets, hospitals must take particular steps to stop the spread. A possible risk to the public’s health and safety is the spread of contagious illnesses from nearby fomites. Inanimate items are breeding grounds for micro-organisms. However, toilet and bathroom door handles and knobs are the most typical cause of fomite infections in most hospitals [2].

Hospitals have always been concerned about the contamination of door handles with multidrug-resistant micro-organisms. The COVID-19 pandemic worsened the problem due to the high infectivity of the SARS-CoV-2 virus [3] and the related increase in antibiotic resistance in bacteria due to the increased use or misuse of antibiotics among COVID-19 patients. According to the Center for Disease Control and Prevention (CDC), multidrug-resistant bacteria are those resistant to multiple drugs, meaning they can withstand at least one antibiotic from three or more drug categories [4]. These types of bacteria are primarily detected in hospitals and healthcare facilities. They are among the most common causes of nosocomial diseases [5].

**Fig. 1.**
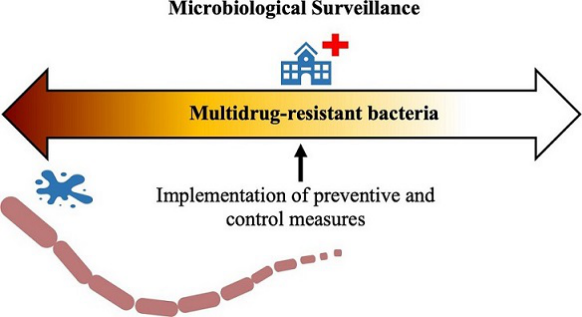
Trend of the prevalence of multidrug-resistant bacteria due to microbial and antimicrobial resistance surveillance.

The transmission of nosocomial diseases through high-contact points in healthcare facilities is a significant concern because these infections can have serious consequences, especially for people who are already sick or have compromised immune systems [6]. Hospitals have taken further steps to address the problem of healthcare-associated infections (HCAIs), such as routine cleaning and disinfection of door handles and other high-touch surfaces, as well as requiring healthcare workers to wear personal protective equipment (PPE). One study found that due to infection prevention programmes necessitated by the pandemic, the prevalence of HCAIs with multidrug-resistant organisms has decreased [7]. We assume that strict prevention protocols have reduced the prevalence of multidrug-resistant bacteria in healthcare facilities. Fig. 1 below represents a summary of our assumption. To see if there has been a reduction in the prevalence of multidrug-resistant bacteria during the pandemic, microbial assessment of high-contact surfaces needs to be performed. Here we focused specifically on door handles. To carry out this assessment, data from this experiment carried out in 2020 were analysed. A similar study by Odigie *et al*. pre-2019 [8, 9] is also used to support our findings.

## Methods

### Study site

The study site was the University of Port Harcourt Teaching Hospital (UPTH) in Rivers State, Nigeria. Ethical clearance was solicited from the university’s research ethics committee; UPTH research ethics committee approval number UPTH/ADM/90/S.II/VOL.XI/840. Ethical approval was obtained from the hospital’s research ethics committee after explaining the rationale for the study, the benefits and the voluntary nature of participation in the research. Permission for the study was sought and obtained from the Head of Departments at the University of Port Harcourt Teaching Hospital. The hospital is one of the two tertiary healthcare institutions in Port Harcourt, the capital of Rivers State, Nigeria. It is a multi-specialist teaching hospital with a 657-bed space offering tertiary, secondary and primary healthcare services. The hospital has an average of 5 toilets in its outpatient clinic for every 12.75 patients and a handashing basin for every 10.63 in-patients, meeting the World Health Organization (WHO) minimum requirements that prescribe 4 or more toilets for outpatient settings and 1 or more for 20 users for inpatient settings [10].

### Study design and period

The study was a descriptive cross-sectional study design. It was a period prevalence survey between December 2019 and March 2020.

### Inclusion criteria

Sample populations were collected from all toilet door handles in the University of Port Harcourt Teaching Hospital.

### Exclusion criteria

Doors in private offices in the University of Port Harcourt Teaching Hospital and doors without handles were excluded.

### Sample collection

Samples were collected aseptically from toilet doors in in-patient wards, clinics, laboratories, administrative offices/pharmacy and public toilets. Samples in our study were collected around noon, which is the hospital’s peak period of patient activity.

A total of 86 samples were collected aseptically using a sterile swab stick moistened with 0.1 % peptone water. Each sample was collected by carefully swabbing the entire surface of the internal and external door handles. Sterile 6 inch sticks were used to wipe the surfaces for ~5 s. The samples were then transported using a cold chain to circumvent the overgrowth of organisms within 1 h of sample collection to the laboratory for analysis.

### Enumeration of the micro-organisms

A 10-fold serial dilution was carried out; 1 ml was taken from stock (swab stick soaked in sterile diluents) using a sterile pipette into 9 ml of sterile 0.1 % peptone water for 10^−2^–10^−6^ dilution. Microbial plating was carried out on a nutrient agar plate and a MacConkey agar plate. The plates were then inverted and incubated at 35 °C for 24 h for nutrient agar and MacConkey agar. After incubation, colonies were counted, and the results were recorded as colony-forming units (c.f.u.) ml^−1^ according to the methods of Public Health England [11]. c.f.u. ml^−1^ were calculated as:

$$c.f.u.\;ml^{-1}=\frac{number\;of\;colonies\;counted\;\times \;dilution\;factor}{volume\;of\;culture\;plated}$$

### Microbiological test

Further identification of isolates was carried out using the following standard biochemical tests [12]: Gram staining, motility test, catalase test, oxidase test, indole test, citrate test, spore staining, methyl red test, Voges–Proskauer (VP) test, triple sugar iron (TSI) agar test and coagulase test.

### Antibiotic susceptibility test

The antibiotic sensitivity pattern was determined by a standardized single-disc method [13]. A colony of the test organism was picked with a sterile wire loop and immersed in peptone water. The turbidity of the suspension was compared against a reference 0.5 McFarland tube. The suspension of the organism was streaked on the entire plate of nutrient agar, and the antibiotic disc (Gram-negative disc for Gram-negative organisms and Gram-positive disc for Gram-positive organisms) was placed on the plate using forceps. The plates were incubated at 37 °C for 24 h. The following commonly-in-use antibiotics were used without any control organism: ceftazidime (CAZ) 30 µg, cefuroxime (CRX) 30 µg, gentamicin (GEN) 10 µg, ciprofloxacin (CPR) 5 µg, ofloxacin (OFL) 5 µg, amoxycillin/clavulanate (AUG) 30 µg, nitrofurantoin (NIT) 300 µg, ceftriaxone (CTR) 30 µg, cloxacillin (CXC) 5 µg and erythromycin (ERY) 15 µg.

### Statistical analysis

SPSS version 23.0 was used to determine the significant difference between the frequencies of occurrence of isolates on the toilet door handles at different UPTH locations. The data for the distribution of bacteria isolates by location are expressed as the mean±standard deviation (sd) and analysed by two-way analysis of variance (ANOVA) followed by Tukey’s post-hoc test. Significance is given as *P*<0.05.

## Results

A total of 329 micro-organisms were isolated, consisting of 253 bacteria (76.9 %) and 76 fungi (23.1 %). Gram-positive bacteria constituted 55.5 % of the isolated bacteria, while 44.4 % were Gram-negative. Table 1 shows the average c.f.u. bacteria counts observed in the different hospital locations. The experiment was carried out in duplicates. The data showed the locations with the highest mean bacteria c.f.u. in the clinics to have a value of 5.64±0.84, while the wards with the lowest mean bacteria c.f.u. had a value of 4.93±0.87.

**Table 1.** Total bacterial counts for door handles at the different UPTH locations presented as mean±standard deviation

Location	Mean c.f.u. ml^−1^ (log_10_)	Minimum c.f.u. ml^−1^ (log_10_)	Maximum c.f.u. ml^−1^ (log_10_)
Ward	4.93±0.50	4.18	5.70
Lab	4.93±0.87	4.11	5.85
Public toilet	5.29±0.48	4.73	5.60
Office area/pharmacy	5.49±0.64	4.60	6.56
Clinic	5.64±0.84	4.45	8.45

**Fig. 2.**
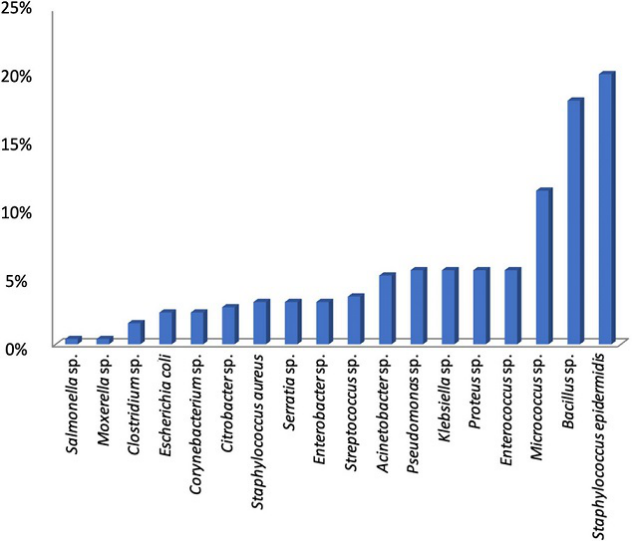
Mean percentage frequency of occurrence of bacterial isolates.

The frequency and prevalence of identified bacteria species are presented in Fig. 2, which shows that *Staphylococcus epidermidis* (20.16 %) and *Bacillus* sp. (18.18 %) had the highest frequency and prevalence. In comparison, *Salmonella* sp. and *Moxorella* sp. (0.40 %) had the lowest frequency and prevalence.

**Fig. 3.**
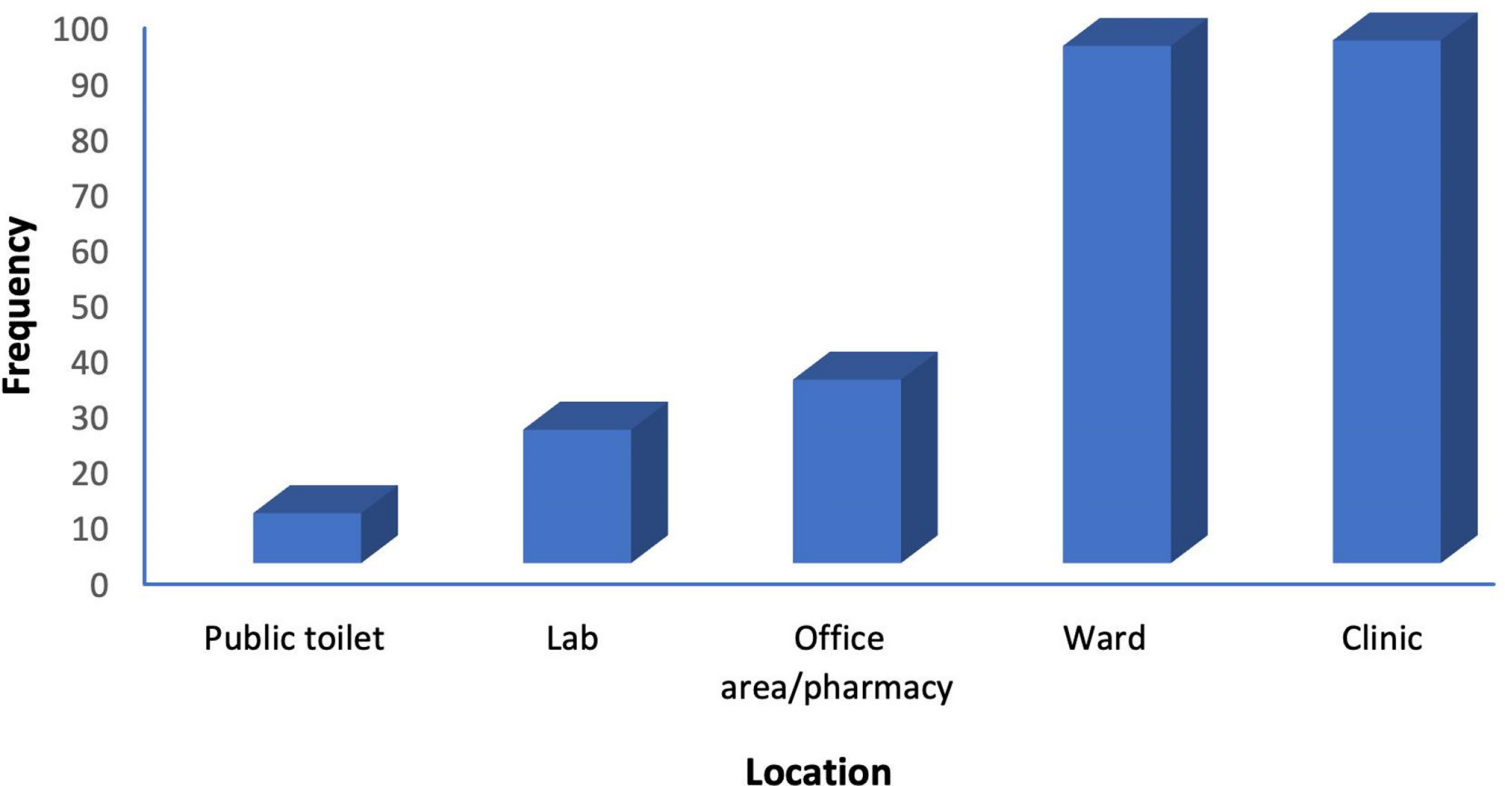
Distribution of bacteria isolated in different locations.

The distribution of bacterial species according to location presented in Fig. 3 shows that 9 (3.56 %) were isolated from the public toilet, 24 (9.49 %) from the laboratories, 33 (13.04 %) from office areas, 93 (36.76 %) from wards and 94 (37.15 %) from clinic areas.

Table 2 shows the distribution of the bacteria isolated in the different areas. The numbers in parentheses represent the percentage isolates. The results are statistically significant (*P*=0.05), meaning that there is a statistical difference between the bacteria isolates and between different locations.

**Table 2.** Distribution of bacterial isolates by location. na, not applicable

Bacteria	Clinic (%)	Ward (%)	Lab (%)	Office area/pharmacy (%)	Public toilet (%)
* Salmonella* sp.	1 (1.06)	na	na	na	na
*Moxorella* sp.	na	na	na	na	1 (0.40)
* Clostridium* sp.	na	2 (2.15)	na	na	2 (0.79)
* Escherichia coli *	2 (2.13)	3 (3.23)	na	1 (3.03)	na
* Acinetobacter* sp.	9 (9.57)	4 (4.30)	na	na	na
* Corynebacterium* sp.	2 (2.13)	1 (1.08)	na	1 (3.03)	2 (0.79)
* Citrobacter* sp.	2 (2.13)	4 (4.30)	na	1 (3.03)	na
* Staphylococcus aureus *	3 (3.19)	2 (2.15)	na	3 (9.09)	na
* Serratia* sp.	1 (1.06)	5 (5.38)	na	2 (6.06)	na
* Enterobacter* sp.	4 (4.26)	3 (3.23)	1 (4.17)	na	na
* Streptococcus* sp.	2 (2.13)	3 (3.23)	2 (8.33)	2 (6.06)	na
* Pseudomonas* sp.	6 (6.38)	5 (5.38)	1 (4.17)	2 (6.06)	na
* Klebsiella* sp.	5 (5.32)	5 (5.38)	2 (8.33)	1 (3.03)	1 (0.40)
* Proteus* sp.	3 (3.19)	7 (7.53)	1 (4.17)	3 (9.09)	na
* Enterococcus* sp.	4 (4.26)	6 (6.45)	2 (8.33)	1 (3.03)	1 (0.40)
* Micrococcus* sp.	13 (13.83)	6 (6.45)	6 (25.00)	2 (6.06)	2 (0.79)
* Bacillus* sp.	18 (19.15)	16 (17.20)	5 (20.83)	7 (21.21)	na
* Staphylococcus epidermidis *	19 (20.21)	21 (22.58)	4 (16.67)	7 (21.21)	na
**Total**	**94** (100.0)	**93** (100.0)	**24** (100.0)	**33** (100.0)	**9** (100.0)
**Mean**± **sd**	**5.88**±**5.84**	**5.81**±**5.29**	**2.67**±**1.87**	**2.54**±**2.11**	**1.50**±**0.55**

Table 3 below shows the percentage resistance of the selected bacteria subjected to susceptibility testing. *Enterococcus* sp. showed multiple resistance to cefixime, ciprofloxacin and ceftazidime. *S. epidermidis* and *Micrococcus* sp. only showed resistance to one antibiotic (cloxacillin). Gram-positive organisms were tested on Gram-positive discs, and Gram-negative organisms on Gram-negative discs. Hence not applicable (na) represents organisms that do not apply to the discs being used.

**Table 3.** Antimicrobial susceptibility pattern of isolated bacteria

Antibiotics	Susceptibility	* Pseudomonas* sp. (*n*=14, %)	* Enterococcus* sp. (*n*=14, %)	* Klebsiella* sp. (*n*=14, %)	* Proteus* sp. (*n*=14, %)	* Micrococcus* sp. (*n*=29, %)	* Bacillus* sp. (*n*=49, %)	* Staphylococcus epidermidis* (*n*=51, %)
GEN	S	11 (78.6)	10 (71.4)	13 (92.9)	14 (100.0)	28 (96.6)	39 (84.8)	46 (90.2)
	I	3 (21.4)	0 (0.0)	1 (7.1)	0 (0.0)	1 (3.4)	7 (15.2)	5 (9.8)
	R	0 (0.0)	4 (28.6)	0 (0.0)	0 (0.0)	0 (0.0)	0 (0.0)	0 (0.0)
CXM	S	12 (85.7)	10 (71.4)	11 (78.6)	14 (100.0)	na	na	na
	I	2 (14.3)	0 (0.00)	3 (21.4)	0 (0.0)	na	na	na
	R	0 (0.0)	2 (28.6)	0 (0.0)	0 (0.0)	na	na	na
CPR	S	12 (85.7)	10 (71.4)	12 (85.7)	14 (100.0)	na	na	na
	I	2 (14.3)	3 (28.6)	2 (14.3)	0 (0.0)	na	na	na
	R	0 (0.0)	1 (7.1)	0 (0.0)	0 (0.0)	na	na	na
CRX	S	10 (71.4)	13 (92.9)	12 (85.7)	14 (100.0)	25 (86.2)	35 (86.2)	42 (76.1)
	I	3 (21.4)	0 (0.0)	2 (14.3)	0 (0.0)	4 (13.8)	11 (13.8)	9 (23.9)
	R	1 (7.1)	1 (7.1)	0 (0.0)	0 (0.0)	0 (0.0)	0 (0.0)	0 (0.0)
NIT	S	10 (83.3)	13 (92.9)	12 (85.7)	14 (100.0)	na	na	na
	I	2 (16.7)	1 (7.1)	2 (14.3)	0 (0.0)	na	na	na
	R	0 (0.0)	0 (0.0)	0 (0.0)	0 (0.0)	na	na	na
CAZ	S	10 (71.4)	10 (71.4)	12 (85.7)	14 (100.0)	26 (89.7)	39 (84.8)	44(86.3)
	I	4 (28.6)	4 (28.6)	2 (14.3)	0 (0.0)	3 (10.3)	7 (15.2)	7(13.7)
	R	0 (0.0)	0 (0.0)	0 (0.0)	0 (0.0)	0 (0.0)	0 (0.0)	0 (0.0)
OFL	S	10 (71.4)	12 (85.7)	13 (92.9)	14 (100.0)	27 (93.1)	36 (78.3)	43 (84.3)
	I	4 (28.6)	2 (14.3)	1 (7.1)	0 (0.0)	2 (6.9)	10 (21.7)	8 (15.7)
	R	0 (0.0)	0 (0.0)	0 (0.0)	0 (0.0)	0 (0.0)	0 (0.0)	0 (0.0)
AUG	S	13 (92.9)	12 (85.7)	13 (92.9)	14 (100.0)	28 (96.6)	39 (84.8)	46 (90.2)
	I	1 (7.1)	2 (14.3)	1 (7.1)	0 (0.0)	1 (3.4)	7 (15.2)	5 (9.8)
	R	0 (0.0)	0 (0.0)	0 (0.0)	0 (0.0)	0 (0.0)	0 (0.0)	0 (0.0)
CTR	S	na	na	na	na	26 (89.7)	39 (84.8)	44 (84.8)
	I	na	na	na	na	3 (10.3)	7 (10.3)	7 (15.2)
	R	na	na	na	na	0 (0.0)	0 (0.0)	0 (0.0)
ERY	S	na	na	na	na	26 (89.7)	39 (84.8)	44 (86.3)
	I	na	na	na	na	3 (10.3)	7 (15.2)	7 (13.7)
	R	na	na	na	na	0 (0.0)	0 (0.0)	0 (0.0)
CXC	S	na	na	na	na	26 (89.7)	39 (84.8)	44 (86.3)
	I	na	na	na	na	2 (6.9)	7 (15.2)	6 (11.7)
	R	na	na	na	na	1 (3.4)	0 (0.0)	1 (2.0)

Gram-positive disc key: CAZ,ceftazidime; GEN, gentamycin; CTR, ceftriaxone; ERY, erythromycin; CXC, cloxacillin; OFL, ofloxacin; AUG, augmentin.

Gram-negative disc key: CPR, ciprofloxacin; NIT, nitrofurantoin; CRX, cefuroxime; CAZ, ceftazidime; GEN, gentamycin; OFL, ofloxacin; AUG, augmentin.

I, intermediate; R, resistantS, sensitive.

Table 4 shows that 4 (28.57 %) *Enterococcus* sp. were multidrug-resistant, 1 (1.96 %) *S. epidermidis* was resistant and 1 (3.45 %) *Micrococcus* sp. showed resistance. At the same time, none of the other isolates was found to be multidrug-resistant.

**Table 4.** Pattern of multidrug resistance in bacterial isolates

Bacteria	Multidrug-resistant		Total (*n*, %)
	Yes (%)	No (%)	
* Pseudomonas* sp.	0 (0.0)	12 (100.0)	**12** (100.0)
* Enterococcus* sp.	4 (28.57)	10 (71.43)	**14** (100.0)
* Klebsiella* sp.	0 (0.0)	14 (100.0)	**14** (100.0)
* Proteus* sp.	0 (0.0)	14 (100.0)	**14** (100.0)
* Micrococcus* sp.	1 (3.45)	28 (96.55)	**29** (100.0)
* Bacillus* sp.	0 (0.0)	46 (100.0)	**46** (100.0)
* Staphylococcus epidermidis *	1(1.96)	50(98.04)	**51** (100.0)

Table 5 shows that the distribution of the multidrug-resistant bacteria according to the different locations was not statistically significant (*P*>0.05). Of the four multidrug-resistant bacteria, two (50.0 %) were found in the laboratory area and the other two (50.0 %) were found in the clinic area.

**Table 5.** Distribution of multidrug-resistant bacteria across the different locations

Locations	Multidrug-resistant bacteria		Chi-square (*P*-value)
	Yes	No	
Public toilet	0 (0.0)	4 (2.27)	6.52 (0.1630)*
Lab	2 (50.0)	19 (10.80)	
Office area/pharmacy	0 (0.0)	22 (12.50)	
Ward	0 (0.0)	65 (36.93)	
Clinic	2 (50.0)	66 (37.50)	
**Total**	**4** (100.00	**176** (100.0)	

*Distribution is not statistically significant (*P*>0.05).

## Discussion

According to our results, the prevalence of bacteria mainly associated with multidrug resistance and HCAIs is low. Such bacteria isolated in our studies are *Klebsiella* sp. [14], *Pseudomonas* sp. [15], *Acinetobacter* sp. [16] and *Enterococcus* sp. [17], which all have a mean frequency of occurrence below 10 %. This study showed that 4 (28.57 %) of the *Enterococcus* sp. were multidrug-resistant, while none of the other isolates were found to be multidrug-resistant. This resistant strain is usually vancomycin-resistant *Enterococcus* [18]. The findings of this study are in contrast to the reports of a similar study by Odigie *et al*. [9], where most of the bacterial isolates were multidrug-resistant. The discrepancies between these findings and our study might be because their study was carried out pre-pandemic, where hospital healthcare practices were probably less strict.

A low prevalence of multidrug-resistant bacteria generally has good effects on public health facilities. One of the most important implications is low antimicrobial resistance. This means fewer bacteria will become multi-antibiotic-resistant, thereby reducing the spread of antibiotic resistance, something public health officials and healthcare professionals are very concerned about, according to a report by the European Centre for Disease Prevention and Control (ECDC) [19]. A 2019 study by Murray *et al.* places *Klebsiella pneumoniae *, *Streptococcus pneumoniae *, *Acinetobacter baumannii* and *Pseudomonas aeruginosa* among the six key bacteria responsible for death due to antimicrobial resistance [20]. Our study found these bacteria to be of low prevalence, with a *Streptococcus* sp. frequency of occurrence <5%. Other implications of the low prevalence of multidrug-resistant bacteria includes more treatment options being available for healthcare providers, lower healthcare costs and improved infection control [21].

The most isolated bacterium was *Staphylococcus epidermidis* (20.16 %), followed by *Bacillus* sp. (18.18 %) and *Micrococcus* sp. (11.46 %). This is anticipated, as it is a major component of the normal flora of the skin and nostrils, which is consistent with the findings of similar studies [22, 23]. This contrasts with the results of Odigie *et al*. [8], who found *Escherichia coli* to be the most frequently (92.1 %) isolated bacterium from door handle. However, *Staphylococcus aureus* from their study had the next highest mean frequency of isolation (87 %). The result of this study is also consistent with Itah and Ben 2004, who reported that *Staphylococcus* species (54.7 %) as the most frequent bacteria isolated in the hospital environment [24].

In our study, *S. epidermidis* showed low resistance and no occurrence of multidrug-resistant species. Bacteria such as *S. epidermidis* most certainly do not cause significant HCAI. This is an indication of the low occurrence of the resistant form despite its high prevalence as compared with the pre-Covid-19 study by Odigie *et al*. [9], where *Staphylococcus* species (*S. pneumonia* and *S. aureus *) were multidrug-resistant, with *S. aureus* being resistant to 50 % of the antibiotics. Based on our study, ciprofloxacin was the most effective antibiotic against all bacterial isolates.

The distribution of multidrug-resistant bacteria in our study locations was also statistically insignificant (*P*>0.05). Of the four multidrug-resistant bacteria, two (50.0 %) were found in the laboratory area, and the other two (50.0 %) were found in the clinic area. The laboratory is where these bacteria were isolated and tested, which is probably why their occurrence was high here. However, the total bacterial count distribution is statistically significant, with the clinic and ward area having a higher bacterial count. The clinic area of the hospital has been reported to be the area with the most human traffic, including patients, patients’ visitors and medical personnel [22, 25, 26]. It has also been reported to account for a considerable occurrence of drug-resistant bacterial growth in previous studies [27, 28].

To stop the spread of illness, hospitals must continue to take precautions to lessen the contamination of door handles and other high-touch surfaces and train staff members and patients on the value of good hand hygiene. At the time of this study, we did not explore the hospital’s cleaning procedure or the frequency of cleaning; therefore, this might influence the prevalence of bacteria on the surfaces over time. However, with the time point of sample collection being noon during peak patient activity, we aimed to mitigate the influence of the hospital’s cleaning practices on the prevalence of the isolated bacteria.

This study’s relatively low occurrence of multidrug-resistant bacteria is likely attributed to the strict COVID-19 protocols at the University of Port Harcourt Teaching Hospital according to the Nigeria Centre for Disease Control (NCDC), in line with the WHO requirements. An update on the COVID-19 infection prevention and control guidelines has been available since January 2023 [29].

Further and continuous studies need to be carried out post-pandemic to maintain public health protocols and practices that mitigate the prevalence of multidrug-resistant micro-organisms. This is particularly important as a 2022 study found data indicating increased HCAIs post-COVID-19 pandemic. This has necessitated the reversion to preventive and control measures to withstand any potential pandemic [30]. A previous 2021 study showed increased COVID-19 HCAI rates since 2021 [31]. This study on the microbial assessment of door handles can therefore be used as a surveillance test to check the sanitary conditions of hospitals during a suspected outbreak and the prevalence of multidrug-resistant micro-organisms. This surveillance method can also be applied in monitoring multidrug-resistant micro-organisms’ occurrence during suspected outbreaks in residential homes for elderly people.

The use of self-cleaning antimicrobial surfaces is a new and improved way to reduce hand contamination and dissemination of nosocomial pathogens within and around health facilities. Such surfaces have proven efficient, for example in the review by Butler *et al*. [32], which described nanocoatings and nanosurfaces with antimicrobial efficacy being employed for medical use [32]. Surface skin antibacterial door push pads and pull pads can be placed on toilet door handles within and around the hospital to reduce the spread of bacteria around the health facility. Automated motion sensor doors will also eliminate the need for door handles; however, this might constitute an economic burden for healthcare facilities, especially in primary healthcare centres in rural communities.
